# Clinical characteristics and outcomes of maintenance hemodialysis patients with COVID-19 during the Omicron wave of the pandemic in Beijing: a single center retrospective study

**DOI:** 10.1186/s12882-024-03575-1

**Published:** 2024-04-22

**Authors:** Yujing Pan, Dan Li, Zhoucang Zhang, Jing Xu, Xinping Sun, Mei Wang, Jiaxiang Ding

**Affiliations:** 1 Nephrology Department, Peking University International Hospital, No. 1 Life Park Road, Life Science Park of Zhongguancun, Changping District, 102206 Beijing, P.R. China; 2 Hemodialysis Center, Peking University International Hospital, 102206 Beijing, P.R. China; 3 Department of Clinical Laboratory, Peking University International Hospital, 102206 Beijing, P.R. China

**Keywords:** Coronavirus disease 2019 (COVID-19), Maintenance hemodialysis (MHD), Omicron variant, Prognosis, Severe acute respiratory syndrome coronavirus-2 (SARS-cov-2)

## Abstract

**Background:**

The clinical manifestations and prognosis of hemodialysis patients with severe acute respiratory syndrome coronavirus-2 (SARS-CoV-2) during the Omicron wave of the pandemic infection were still unclear. This study investigated the clinical characteristics of patients undergoing maintenance hemodialysis (MHD) infected with it.

**Methods:**

This retrospective single-center study included 151 patients undergoing MHD. Healthcare workers were selected as control group were assessed from December 1, 2022 to March 31, 2023. Clinical data, laboratory test results, treatment protocols, and prognoses were collected and analyzed.

**Results:**

The study population included 146 patients with MHD, 93 (63.7%) of whom were infected with SARS-CoV-2. The number of non-severe, severe, and critical cases was 84 (90.3%), 4 (4.3%), and 5 (5.3%), respectively. Six patients (6.5%) died during the study period. The main symptoms of SARS-CoV-2 infection, including fever, cough, and fatigue, were less common in patients with MHD than the controls. During SARS-CoV-2 infection, the C-reactive protein (2.9 vs. 11.8 mg/dl, *p* < 0.0001) and ferritin levels(257.7 vs. 537 ng/l, *p* < 0.0001) were elevated. The hemoglobin(113vs 111 g/L, *p* = 0.0001) and albumin levels(39.4 vs. 36.1 g/L, *p* < 0.0001) decreased. Generally, it took two months for the hemoglobin levels to recover. Positivity rate for SARS-COV-2 serum immunoglobin G (IgG) antibodies and IgG titers were lower in dialysis patients than the controls. Age was positively associated with disease severity, while age and hyponatremia were associated with death.

**Conclusions:**

Patients with MHD and COVID-19 were primarily classified as non-severe. SARS-CoV-2 infection would soon lead to the increase of inflammation related acute response protein in dialysis patients, and then lead to the decrease of hemoglobin and albumin. About 9.6% in HD patients were severe cases and had poor prognosis. Advanced age and hyponatremia were associated with disease severity and prognosis.

**Supplementary Information:**

The online version contains supplementary material available at 10.1186/s12882-024-03575-1.

## Background

Since the first case of coronavirus disease 2019 (COVID-19) in December 2019, hundreds of millions of people have been infected with severe acute respiratory syndrome coronavirus-2 (SARS-Cov-2) worldwide. Maintenance hemodialysis (MHD) patients are at a much higher risk of COVID-19 than the general population and have a nearly 20% mortality rate. This is attributed to the dysfunction of both the innate and adaptive immune systems and the number of underlying comorbid diseases in MHD patients [1]. Many studies have previously established the clinical features of hemodialysis patients with COVID-19. Approximately 5.5% of patients undergoing MHD have developed COVID-19 [2]. In the US, mortality rate is exceeding 20% in dialysis patients with COVID-19 [3]. Chen et al. conducteda meta-analysis of 396,062 patients undergoing hemodialysis (HD). The incidence of COVID-19 in these patients was 7.7% (95% CI: 5.0–10.9%). The overall mortality rate was 22.4% (95% CI:17.9–27.1%). The reported estimates are higher in non-Asian countries than in Asian countries [4]. However, a few highly mutated variants of SARS-CoV-2 like the omicron variant which were extremely transmissible, and could evade the immune system, have raised global concern. Since the epidemic of COVID-19 in Beijing in December 2022, the Omicron variant and BF.7 variant along with its descendant lineages have been predominant [5]. The clinical characteristics of patients with MHD infected with SARS-CoV-2 during the omicron epidemic are unclear. Therefore, in this study, we aim to elucidate the clinical characteristics of MHD patients infected with SARS-CoV-2 during the omicron epidemic.

## Methods

### Study population

This retrospective study focused on the clinical characteristics of confirmed COVID-19 cases at the hemodialysis center of Peking University International Hospital. The study participants which were recruited from December 1,2022 to March 30,2023 were divided into two groups: all MHD patients and healthcare workers as control group. Patients who did not receive at least three months of outpatient dialysis at the time of infection were excluded.

### Study definition

COVID-19 was defined according to the World Health Organization (WHO) Clinical management: living guidance. The diagnosis of COVID-19 was based on a positive result on nucleic acid amplification test (NAAT), or a rapid antigen test of nasal and/or throat swab specimens, or a positive result for immunoglobulin M or G antibodies in unvaccinated patients.

The COVID-19 cases were classified based on the WHO Clinical management: living guidance. It was classified as a severe disease if the blood oxygen saturation was < 90% under indoor air conditions or if showing the signs of pneumonia and severe respiratory distress. It was classified as a critical disease if one of the following was present: acute respiratory distress syndrome (ARDS), sepsis, septic shock, or requiring life-sustaining treatment (e.g., invasive or noninvasive mechanical ventilation or vasopressor therapy). Non-severe COVID-19 was defined as not meeting any of the criteria for severe or critical disease. Infection rate was defined as the number of active COVID-19 cases divided by the number of patients requiring dialysis. Mortality was defined as the number of deaths divided by the total number of COVID-19 cases. Hospitalization was defined as any hospitalization that occurred within two weeks of SARS CoV-2 diagnosis [6]. Asymptomatic patients were diagnosed in those in whom symptoms were not obviously. The time to symptom resolution was calculated from the date when any of the symptoms including fever, cough, fatigue and so on was noticed by the patients to the date when all symptoms had disappeared. The virus shedding time indicated the duration from the first positive antigens or nucleic acids result to the first consecutive negative antigens or nucleic acids result.

### SARS-COV-2 nucleic acid and serum antibody measurement

NAAT for SARS-CoV-2 was done using a quantitative real-time reverse transcriptase–polymerase chain reaction (RT-PCR) assay of nasal or throat swab specimens. The cutoff of cycle threshold (Ct) value for nucleic acid detection test was set at 38 according to the manufacturer’s instructions (Beijing Zhuo Cheng Hui Sheng Biotechnology Co., Ltd., Beijing, China). In total, 139 of 146 MHD patients were tested for SARS-COV-2 serum antibody. Serum immunoglobin M (IgM) and immunoglobin G (IgG) antibodies against SARS-CoV-2 were detected using a magnetic particle chemiluminescence immunoassay. The antibodies were captured based on the method supplied by Mike Biotechnology, Inc., according to the manufacturer’s instructions. The assay is primarily directed against the SARS-CoV-2 nucleocapsid (N) protein, with some reactivity towards the spike (S)protein. The antibody levels were expressed using the relative binding signals compared to the cutoff value of each assay (S/CO). The value of S/CO beyond 1.0 indicated a positive result for immunoglobulin M or G antibodies.

### Data collection

Data, including demographic information, medical history, exposure history, comorbidities, symptoms, signs, laboratory examinations, chest computed tomography (CT) scans, and treatment measures, were obtained from the electronic medical record system of Peking University International Hospital. A trained physician and a nurse on the research team collected the epidemiological and symptom data and double-checked the data from the electronic medical record system.

### Statistical analyses

Quantitative variables were reported as mean ± SD, and frequency (%) was used to express categorical variables. An independent t-test was used to compare the quantitative variables between the two groups. Chi-square and Fisher’s exact tests were used to compare the qualitative variables between the two groups. The Shapiro–Wilk test was used to verify whether the clinical data were normally distributed. Comparisons among the three groups were conducted using repeated-measures ANOVA or the Kruskal-Wallis test. Data analysis was performed using SAS v9.4 (SAS Institute), and statistical significance was set at *P* value less than 0.05.

## Results

**Fig. 1:**
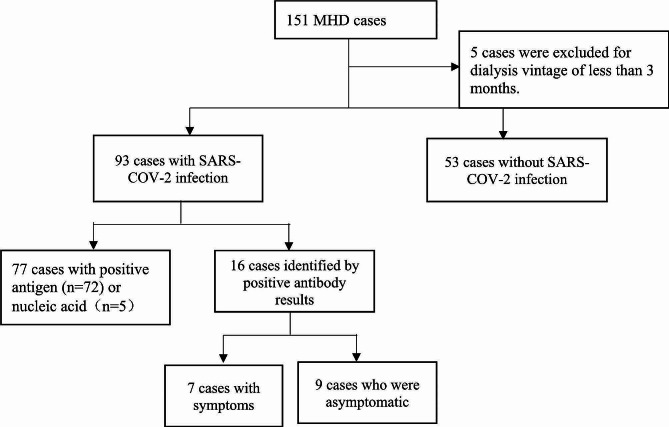
Diagnosis flow chart

There were 151 MHD patients in our center, of which five patients were excluded owing to dialysis vintage being less than 3 months. Among the 146 patients, 93 (63.7%) were infected with SARS-CoV-2 based on positive antigen, nucleic acid, or antibody results. Among these 93 patients, 77 tested positive for antigens or nucleic acids, and 16 tested positive for antibodies. Among the 53 patients without SARS-COV-2 infection, only one patient was tested for antigens; the rest were not tested for antigens or nucleic acids. A diagnostic flowchart of the study is shown in Fig. 1.

### Clinical characteristics of MHD patients with COVID-19

The incidence of SARS-CoV-2 infection in our hemodialysis center was 63.7%, which was lower than that in the control group. When comparing patients with COVID-19 and those without COVID-19, we noted a median age of 60 years (IQR 47, 68 years), and the number of male patients was 57 (61.3%). The median duration of hemodialysis was 65 months (IQR 43,92 months) in patients with COVID-19. Patients with COVID-19 had higher CRP (11.8 vs. 5.2 mg/dl), ferritin level (537 vs. 386.2 ng/l) and hyponatremia (136 vs. 138 mmol/l) levels. No significant differences in age, sex, primary kidney disease, coexisting disorders, average pre-HD blood pressure, the urea clearance index (Kt/V), or vaccination rates were found between the SARS-CoV-2 infected and uninfected group (Table 1). The time to symptom resolution in HD patients were 10 (IQR 4,20) days, and the viral shedding time was 7 (IQR 7,10) days. The time to symptom resolution in controls was 10 (IQR 7,14) days and the viral shedding time was 7 (IQR 7,8) days. No significant differences in the time of symptom resolution (*p* = 0.4) or viral shedding time (*p* = 0.09) were found between patients with COVID-19 and controls. There was no significant difference in the baseline characteristics between asymptomatic and symptomatic patients with COVID-19 (Supplementary data, Table S1). There was no significant difference in laboratory indicators between asymptomatic and symptomatic patients with COVID-19 during SARS-CoV-2 infection ((Supplementary data, Table S2).

**Table 1:** Baseline characteristics in MHD patients with or without COVID-19

	COVID19-infected patients *N* = 93	Non-infected patients *N* = 53	*P*
Age (yrs, median and IQR)	60(47,68)	57(45,67)	0.5
Dialysis vintage (mean, median and IQR)	65(43,92)	68(43,95)	0.7
Male sex (No, %)	57(61.3)	38(71.7)	0.2
Vaccinations (No, %)	16(17.2)	7(13.2)	0.7
Primary disease (No,%)			0.97
Chronic glomerulonephritis	35(38.5)	20(37.8)	
Diabetic nephropathy	26(28.6)	15(28.3)	
Hypertensive nephropathy	8(8.8)	6(11.3)	
Others	22(24.2)	12(22.6)	
Coexisting disorders (No,%)			
Diabetes mellitus	32(35.2)	20(37.7)	0.8
Cancer	3(3.3)	0	0.3
BMI (kg/m2, median and IQR)	24.0(21.7,26.7)	24.1(22.2,27.5)	0.6
Monthly averaged pre-HD systolic BP (mmHg, mean ± SD)	152.5 ± 16.0	153.4 ± 19.4	0.8
Monthly averaged pre-HD diastolic BP (mmHg, mean ± SD)	79.3 ± 12.5	81.0 ± 13.0	0.4
KT/V (median and IQR)	1.5(1.3,1.7)	1.4(1.3,1.7)	0.3
Weekly averaged EPO dose (IU, median and IQR)	9000(6000,12000)	6000(6000,9000)	0.2
Hemoglobin (g/L, median and IQR)	111(106,118)	113(106,119)	0.5
Albumin (g/L, mean ± SD)	36.1 ± 3.4	36.7 ± 3.1	0.36
Potassium (mmol/L, mean ± SD)	4.4 ± 0.6	4.4 ± 0.6	0.98
Sodium (mmol/L, mean ± SD)	135.5 ± 3.6	137.6 ± 2.4	< 0.0001
Ferritin (ng/L, median and IQR)	537.0(340.2,814.6)	386.2(185.0,650.8)	0.006
Serum iron (umol/L, median and IQR)	9.4(6.7,12.9)	10.2(8.3,11.9)	0.3
Leukocyte (10^9/l, median and IQR)	5.5(4.3,6.8)	6.1(4.5,7.5)	0.43
Lymphocyte (10^9/l, median and IQR)	0.9(0.7,1.2)	0.9(0.8,1.2)	0.8
C-reactive protein (mg/l, median and IQR)	11.8(3.3,43.7)	5.2(2.3,11.8)	0.005
ALT (IU/L, median and IQR)	10.0(8.0,18.0)	10.0(7.0,14.0)	0.6
AST (IU/L, median and IQR)	17.0(12.0,23.0)	13.0(11.0,17.0)	0.003
ALP (IU/L, median and IQR)	84.0(67.0,118.0)	89.0(68.0,111.0)	0.9

Abbreviations: ALT, alanine aminotransferase; AST, aspartate aminotransferase; ALP, alkaline phosphatase

**Fig. 2:**
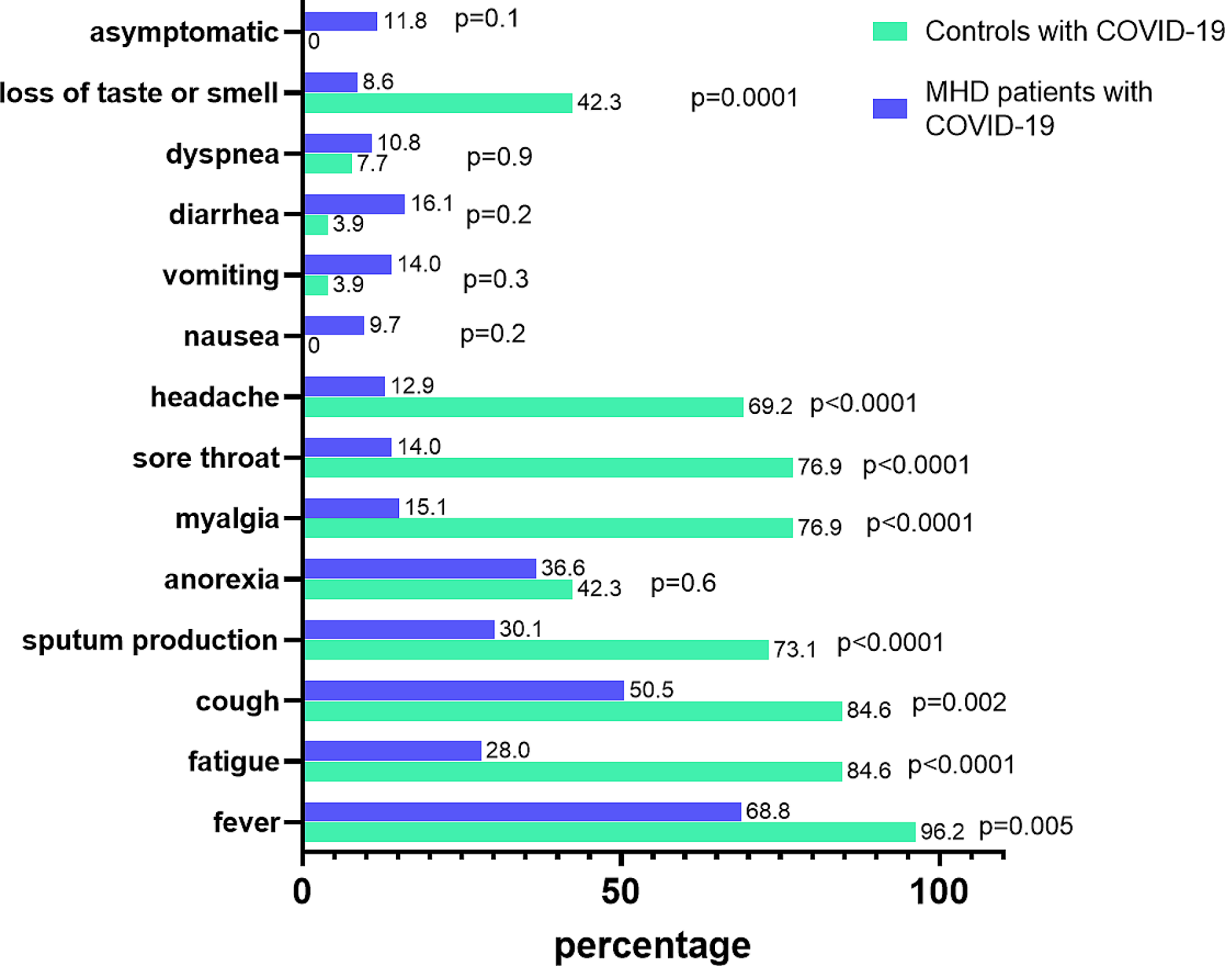
Common signs and symptoms of MHD patients and controls with COVID-19

Fever (68.8%) and cough (50.5%) were the most common symptoms in patients with COVID-19. Other symptoms included fatigue (28%), sputum production (30.1%), diarrhea (16.1%), vomiting (14%), and headache (12.9%). Fever (96.2%), coughing (84.6%), and fatigue (84.6%) were the most common symptoms in the controls with COVID-19 (Fig. 2). The main symptoms of COVID-19, including fever, cough, and fatigue, were less common in patients receiving MHD. Moreover, 11.8% of MHD patients showed no obvious COVID-19 symptoms. During SARS-CoV-2 infection, C-reactive protein (CRP) and ferritin levels were elevated while the hemoglobin, albumin, serum potassium, and sodium levels decreased. A significant decrease in hemoglobin levels occurred one month after the SARS-CoV-2 infection, and with the control of SARS-CoV-2 infection, CRP, potassium, and albumin gradually returned to normal after one month, whereas it took two months for the hemoglobin levels to recover (Table 2). The hemoglobin levels increased to 112 (105,116) g/l after two months. However, the weekly average EPO dose remained higher than the pre-infection dose. (Supplementary data, Table S3).

**Table 2:** Changes after SARS-CoV-2 infection in MHD patients

	Before SARS-CoV-2 infection	During SARS-CoV-2 infection	After SARS-CoV-2 infection one month	*P*
Monthly averaged pre-HD systolic BP (mmHg, mean ± SD)	151.4 ± 15.4	152.5 ± 16.0	150.4 ± 17.1	0.3
Monthly averaged pre-HD diastolic BP (mmHg, mean ± SD)	78.4 ± 11.6	79.3 ± 12.5	78.6 ± 11.7	0.5
KT/V (median and IQR)	1.5(1.4,1.7)	1.5(1.3,1.7)	1.5(1.4,1.7)	0.9
Weekly averaged EPO dose (IU, median and IQR)	7500(6000,9000)	9000(6000,12000)	9000(6000,12000)	0.053
Hemoglobin (g/L, median and IQR)	113.0(108.0,120.0)	111.0(106.0,118.0)	107.0(99.0,115.0)	0.0001
Albumin (g/L, mean ± SD)	39.4 ± 3.2	36.1 ± 3.4	38.5 ± 4.5	< 0.0001
Potassium (mmol/L, mean ± SD)	4.8 ± 0.7	4.4 ± 0.6	4.6 ± 0.6	< 0.0001
Sodium (mmol/L, mean ± SD)	136.5 ± 2.9	135.5 ± 3.6	135.5 ± 3.7	0.0007
Ferritin (ng/L, median and IQR)	257.7(171.0,361.8)	537.0(340.2,814.6)	-	< 0.0001
Serum iron (umol/L, median and IQR)	10.3(8.9,12.9)	9.4(6.7,12.9)	-	0.02
Leukocyte (10^9/l, median and IQR)	6.2(4.8,7.0)	5.5(4.3,6.8)	6.3(4.9,7.9)	0.035
Lymphocyte (10^9/l, median and IQR)	1.1(0.8,1.4)	0.9(0.7,1.2)	1.1(0.8,1.3)	0.052
C-reactive protein (mg/l, median and IQR)	2.9(1.6,9.1)	11.8(3.3,43.7)	5.6(2.6,14.7)	< 0.0001
Predialysis Weight (kg, median and IQR)	67.7(57.6,77.3)	66.2(56.9,77.0)	66.1(56.9,77.2)	0.9

### Clinical outcomes of MHD patients with COVID-19

During the COVID-19 epidemic, 93 (63.7%) patients on MHD were infected with SARS-CoV-2 and 12 patients were admitted to the hospital. The mortality rate was found to be 6.5% and two deaths occurred two months after SARS-CoV-2 infection. However, only two mortality was directly attributed to COVID-19,while two were caused by severe pneumonia and one by acute cerebral infarction and the other by gastrointestinal bleeding. Four (4.3%) patients with blood oxygen saturation < 90% were classified as severe and another five (5.3%) were classified as critical. Most patients with non-severe disease receive symptomatic treatment, including nonsteroidal anti-inflammatory drugs and proprietary herbal medicines. Among the non-severe patients, two patients were given antiviral therapy. One patient received Azivudine at a dose of 2 mg on day 1 and 3 mg on day 2, while the other patient received Paxlovid at a dose of 150 mg nirmatrelvir (with 100 mg ritonavir) twice daily for 7 days. All severe patients and two critical patients were administered glucocorticoids at a dosage of 40 mg methylprednisolone or 5–10 mg dexamethasone for 5–10 days. One of the critical patients received a dose of 300 mg nirmatrelvir (with 100 mg ritonavir), followed by a daily dose of 150 mg after dialysis on dialysis days for 5 days. All surviving patients recovered and were eventually discharged.

### Serologic profile of patients with SARS-CoV-2 infection

In total, 46 of the 93 patients presented exclusively with IgG antibodies, one patient presented with IgM antibodies, and eight patients had both IgM and IgG antibodies. The IgG positivity rate and IgG titers were lower in patients on dialysis than the controls (Table 3). Among MHD patients with COVID-19, 16 (17.2%) patients had been vaccinated. Out of those vaccinated, 11 (11.8%) patients had received two-dose vaccination, while 5(5.4%) patients had received one-dose vaccination. The patients were vaccinated with inactivated SARS-CoV-2 vaccines produced by Sinovac Biotech and Sinopharm.

**Table 3:** Comparison between MHD patients and controls with COVID-19

	COVID-19-infected patients (*N* = 93)	COVID19-infected controls ( *N* = 26)	*P*
No. of Vaccinations	16(17.2%)	21(80.8%)	< 0.0001
IgG positivity rate	54(58.1%)	21(80.8%)	0.03
IgM positivity rate	9(9.7%)	1(3.9%)	0.58
IgG titers	1.4(0.7,5.6)	11.5(10.5,11.7)	< 0.0001
IgM titers	0.14(0.08,0.3)	0.08(0.05,0.11)	0.0007
IgG titers (one month later)	1.4(0.7,6.0)^a^		
IgM titers (one month later)	0.08(0.05,0.2)^b^		

Note: No significant difference in IgG titers was found at two months in patients with COVID-19(^a^*p*=0.8). IgM titers decreased after one month later (^b^*p*=0.0007)

### Characteristic associated with mortality

Univariate logistic regression analysis revealed the risk factor for severe COVID-19: advanced age (Table 4). The variables identified as significant for mortality included advanced age and hyponatremia (Table 5). No independent association was observed between laboratory features(such as hemoglobin, albumin and CRP) and disease severity.

**Table 4:** Univariate logistic regression analysis of clinical and laboratory features against severe disease

Variables	non-severe COVID-19 *N* = 84	Severe COVID-19 *N* = 9	*P*	OR^a^(95%CI)
Clinical features				
Age (yrs, median and IQR)	59(47,67.5)	74(68,78)	0.0009	1.14(1.05,1.25)
Male sex(No, %)	50(59.5)	7(77.8)	0.5	2.38(0.45,12.16)
Dialysis vintage (mean, median and IQR)	65(43.5,91)	61(43.5,124)	0.9	1.01(0.99,1.02)
Vaccinations (No,%)	14(16.7)	2(22.2)	1	1.43(0.27,7.61)
Diabetes mellitus(No,%)	29(34.5)	5(55.6)	0.4	2.37(0.59,9.51)
Obesity^b^ (No,%)	17(20.2)	1(11.1)	0.8	0.49(0.06,4.21)
Laboratory features				
Albumin (g/L, mean ± SD)	39.4 ± 3.2	39.2 ± 3.7	0.9	0.99(0.80,1.22)
CRP (mg/l, median and IQR)	2.9(1.6,9.2)	2.0(1.3,6.5)	0.3	0.95(0.84,1.08)
Hemoglobin(g/L, median and IQR)	113(108,120)	111(106,117)	0.7	0.99(0.92,1.06)
Sodium (mmol/L, mean ± SD)	136.6 ± 3.0	135.7 ± 2.2	0.4	0.90(0.71,1.14)

^a^OR: odds ratio;

^b^obesity is defined as body mass index ≥ 28 kg/m^2^

**Table 5:** Univariate logistic regression analysis of clinical and laboratory features against mortality

Variables	Alive *N* = 87	Died *N* = 6	*P*	OR^a^(95%CI)
Clinical features				
Age (yrs, median and IQR)	59(47,68)	72.5(67,78)	0.02	1.11(1.01,1.3)
Male sex(No, %)	51(58.6)	6(100)	0.1	
Dialysis vintage (mean, median and IQR)	65(42,93)	72(59,83)	0.7	0.99(0.98,1.02)
Vaccinations (No,%)	14(16.1)	2(33.3)	0.6	2.61(0.43,15.6)
Diabetes mellitus(No,%)	30(34.5)	4(66.7)	0.3	3.80(0.66,21.96)
Obesity^b^(No, %)	17(19.5)	1(16.7)	1	0.82(0.09,7.52)
Laboratory features				
Albumin (g/L, mean ± SD)	39.5 ± 3.1	37.7 ± 5.1	0.4	0.84(0.66,1.08)
CRP (mg/l, median and IQR)	2.8(1.6,8.9)	7.3(0.6,10.3)	0.8	1.02(0.99,1.05)
Hemoglobin(g/L, median and IQR)	113(108,120)	117.5(107,126)	0.5	1.04(0.96,1.13)
Sodium (mmol/L, mean ± SD)	136.6 ± 2.9	134 ± 3.0	0.03	0.73(0.54,0.99)

^a^OR: odds ratio;

^b^obesity is defined as body mass index ≥ 28 kg/m^2^

## Discussion

In our study, we found that MHD patients infected with SARS-CoV-2 were mainly of the non-severe type, with a low proportion of severe and critical types. The main symptoms of COVID-19 were less common in patients on MHD, which may cause difficulties in early diagnosis. During SARS-CoV-2 infection, inflammatory indicators are elevated, and hemoglobin and albumin levels decrease. However, it took longer for hemoglobin levels to recover. Positivity rate for SARS-COV-2 serum IgG antibodies and IgG titers were lower in dialysis patients than in controls. Advanced age were positively associated with disease severity, while advanced age and hyponatremia were associated with death.

Fever and cough were the most common symptoms in hemodialysis patients with COVID-19. A study in Wuhan found that cough (69.7%) and fever (37.9%) were the most common symptoms in hemodialysis patients with COVID-19 [7]. Compared to the patients infected with the delta variant, no major differences in the initial clinical symptoms were identified in patients infected with the Omicron variant [8]. The Omicron variant accounted for the majority of the 6th wave of COVID-19 cases in Japan. In this study, sore throat was more frequent in the 6th wave group (60.4%) than in the 1st–5th wave groups (10.6%; *p* < 0.0001) [9]. In our study, sore throat was the least common symptom (14%). However, the main symptoms of COVID-19, including fever, cough, and fatigue, were found to be less common in patients with MHD than in controls, and may cause difficulties in early diagnosis. Immune disorders involving both the innate and adaptive responses are common in patients with end-stage renal disease undergoing chronic hemodialysis [1]. Therefore, patients with MHD may present atypical features.

Based on the laboratory parameters, hemoglobin and albumin levels decreased. A poor erythropoietin response in inflammatory states and malnutrition leads to anemia. Hypoproteinemia results from inadequate food intake and inflammation. However, it takes a longer (2months) for the hemoglobin levels to recover. Haruta et al. found that CRP levels were significantly higher in the 1st–5th wave group [8.14 (2.84–11.24] mg/dL) than in the 6th wave group [1.99 (0.59–6.00) mg/dL, *p* < 0.001) [9]. On admission to the hospital, the C-reactive protein level in patients with the omicron variant was 1.57 (0.14–4.20) mg/L [8]. However, we found that patients with SARS-CoV-2 infection had higher CRP levels [11.8(3.3–43.7) mg/dl).

In the 6th Japanese wave of COVID-19, the prognosis of hemodialysis patients was good. Critical disease was observed in 21.3% of patients in the 1st to 5th wave group and in 0% in the 6th wave group [9]. Advances in vaccination and treatment may have contributed to these outcomes. However, several other studies have reported conflicting results. Among adults admitted to the hospital with COVID-19, the omicron variant was associated with less severe disease than the delta variant but still resulted in substantial morbidity and mortality. Patients with the omicron variant were found to be at risk of critical illness and death, with 7% of the patients infected with the omicron variant dying in the United States and 15% of patients requiring invasive mechanical ventilation [10]. Chimon et al. found that the case fatality rate was 5% and severe forms of infection were observed in 14% of patients, suggesting that hemodialysis patients remain at a high risk of severe complications after SARS-CoV-2 Omicron infection [11]. During the fifth Omicron wave in Hong Kong, the mortality attributable to COVID-19 in the hemodialysis population was 2.2 deaths per 100 dialysis patients [12]. The mortality rate of our HD patients was 6.5 deaths per 100 patients on dialysis. Bao et al. found that in 102 hemodialysis patients, 12 (11.8%) died [13]. Wen found that the mortality was 5.7% which was similar with our study [14]. The omicron variant was associated with less severe disease than the delta variant but still resulted in substantial morbidity and mortality, especially among hemodialysis patients. Hence, vaccination rates should be improved, and treatment advances should be made.

Antibody testing has been suggested to confirm infections caused by many known pathogenic viruses. Normally, the IgG antibody response in serum lasts longer and indicates a past infection, whereas IgM may represent a recent infection [15]. Recent studies have found that a serological response is detectable 9 days or later after symptom onset; the rates of seropositivity were 94% for anti-nucleoprotein (NP) IgG, 88% for anti-NP IgM, 100% for anti-receptor binding domain (RBD) IgG, and 94% for anti-RBD IgM [16]. Serological assays complement real-time polymerase chain reaction for diagnosis [16, 17]. In our study, serological assays were performed at one and two months. Sixteen patients were identified by positive antibody results, of which three were negative for antigens and the rest were not tested for antigens. Nine of 16 patients were asymptomatic. IgG titers did not change after 2 months of infection. Serological tests revealed asymptomatic SARS-CoV-2 infection and provided further information regarding the full spectrum of the disease in patients with MHD.

A previous study in Shanghai found that full vaccination was a significant protective factor against severe infections (0.237 [0.071–0.793], *p* = 0.019) in patients aged > 60 years [18]. Wing et al. found that a three-dose mRNA COVID-19 vaccination was associated with a lower incidence of SARS-CoV-2 infection and lower severe SARS-CoV-2-related outcomes during the Omicron wave than two doses [19]. mRNA vaccines were found to be highly effective in preventing COVID-19 associated hospital admissions related to the alpha, delta, and omicron variants; however, three vaccine doses were required to achieve protection against omicron variant, similar to the protection provided by two doses against the delta and alpha variants [10]. However, the vaccination rate at our center was very low. The IgG positivity rate and IgG titers were lower after omicron variant infection in patients with MHD than in controls, probably because of low vaccination rates and dysfunction of both the innate and adaptive immune systems. The high rate of infection among controls may have been due to early vaccination. All controls received a third vaccine in 2021. Early and careful preventive steps, including vaccination, are key to suppressing Omicron infections.

Similar to the previous studies, older patients tended to show a poorer prognosis [14, 20]. Several previous studies have found that hyponatremia was associated with an increased death risk in MHD patients [21–23]. Hyponatremia among COVID-19 was also found to be significantly associated with increased odds for mortality (OR = 1.97[95%CI,1.50–2.59]) [24]. This review demonstrated that the most common mechanisms of hyponatremia reported among SARS-COV-2 patients are the syndrome of anti-diuretic hormone secretion(SIADH), followed by adrenal causes, then hypovolemia. When exploring these mechanisms, they are found to be interrelated. In our study, we found that hyponatremia was associated with disease severity and prognosis in MHD patients with COVID-19. Serum sodium levels are influenced by dialysate and dietary sodium, as well as fluid intake in patients with MHD [22]. The underlying mechanisms that lead to hyponatremia in MHD patients with COVID-19 are multifactorial involving factors like gastrointestinal losses, and dietary sodium decreases. Additionally, hyponatremia associated with congestive heart failure, which can occur at different stages in the course of COVID-19 [25]. This can be attributed to non-osmotic release of arginine vasopressin and reduced free water clearance by the kidney [26]. Congestive heart failure can contribute to hyponatremia in MHD patients with residual kidney function [27]. There are several mechanisms by which hyponatremia may directly predispose to mortality among hemodialysis patients. For instance, hyponatremic patients may experience fluctuations in their serum sodium and osmolarity due to variations in dialysate sodium concentrations, which can be detrimental [22]. Moreover, hyponatremia may have direct toxic to other end organs including the brain [28], heart [29]and musculoskeletal system [30].

This study has some limitations. First, because of the limited number of cases from a single center, it was difficult to evaluate the risk factors for disease severity and mortality using multivariable-adjusted methods, and the possible lack of generalizability to other dialysis populations. A multicenter cohort study would help define the clinical manifestations, risk factors, and outcomes. Second, false-positive and false-negative results may be obtained for antigen, nucleic acid, and antibody tests.

## Conclusions

In summary, patients with MHD and COVID-19 were primarily classified as non-severe. SARS-CoV-2 infection would soon lead to the increase of inflammation related acute response protein in dialysis patients, and then lead to the decrease of hemoglobin and albumin. About 9.6% in HD patients were severe cases and had poor prognosis. Advanced age and hyponatremia were associated with disease severity and prognosis.

### Electronic supplementary material

Below is the link to the electronic supplementary material.

Supplementary Material 1

## Data Availability

The data included in this article will be shared upon reasonable request from the corresponding authors.

## Abbreviations

ALT: Alanine aminotransferase
AST: Aspartate aminotransferase
ALP: Alkaline phosphatase
ARDS: Acute respiratory distress syndrome
BMI: Body mass index
CI: Confidence interval
COVID-19: Coronavirus disease 2019
CRP: C- reactive protein
CT: Computed tomography
IgM: Immunoglobin M
IgG: Immunoglobin G
IQR: Interquartile range
Kt/V: The urea clearance index
MHD: Maintenance hemodialysis
NAAT: Nucleic acid amplification test
OR: Odds ratio
RT-PCR: Reverse transcriptase–polymerase chain reaction
SARS-CoV-2: Severe acute respiratory syndrome coronavirus-2
SIADH: Syndrome of anti-diuretic hormone secretion
WHO: World Health Organization
