# Supplementary material for: Clinical characteristics and outcomes of maintenance hemodialysis patients with COVID-19 during the Omicron wave of the pandemic in Beijing: a single center retrospective study
Source: BMC Nephrol. 2024 Apr 22;25:143. doi: 10.1186/s12882-024-03575-1 (PMC11036696; doi:10.1186/s12882-024-03575-1)
Supplement: Supplementary file 1 — Supplementary Material 1 [file 12882_2024_3575_MOESM1_ESM.docx]

Supplementary Material

Supplemental Table S1. Baseline characteristics of asymptomatic patients and symptomatic patients with COVID-19

|  | Asymptomatic patients  N=11 | Symptomatic patients  N=82 | | P |
| --- | --- | --- | --- | --- |
| Age (yrs, median and IQR) | 63(48,69) | 59(47,68) | 0.6 | |
| Dialysis vintage (mean, median and IQR) | 66(45,135) | 65(43,90) | 0.8 | |
| Male sex (No, %) | 5(45.5) | 52(63.4) | 0.4 | |
| Vaccinations (No, %) | 0 | 16(19.5) | 0.2 | |
| Coexisting disorders (No, %) |  |  |  | |
| Diabetes mellitus | 5(45.5) | 29(35.4) | 0.7 | |
| Cancer | 0 | 3(3.7) | 1.0 | |
| BMI (kg/m2, median and IQR) | 28.6(23.1,30.9) | 24(21.9,26.4) | 0.1 | |
| Monthly averaged pre-HD systolic BP (mmHg, mean ± SD) | 154.0±19.3 | 151.1±14.9 | 0.5 | |
| Monthly averaged pre-HD diastolic BP (mmHg, mean ± SD) | 80.5±16.1 | 78.1±11.0 | 0.8 | |
| KT/V (median and IQR) | 1.5(1.3,1.8) | 1.5(1.4,1.7) | 0.7 | |
| Weekly averaged EPO dose (IU,median and IQR) | 9000(6000,15000) | 7500(6000,9000) | 0.2 | |
| Hemoglobin (g/L,median and IQR) | 113(100,121) | 113(108,120) | 0.6 | |
| Albumin (g/L, mean ± SD) | 38.2±3.4 | 39.6±3.2 | 0.2 | |
| Potassium (mmol/L, mean ± SD) | 4.8±0.6 | 4.8±0.7 | 0.9 | |
| Sodium (mmol/L, mean ± SD) | 136.3±3.3 | 136.5±2.9 | 0.8 | |
| Ferritin (ng/L, median and IQR) | 280.8(134.0,379.6) | 257.7(171.0,361.8) | 0.9 | |
| Serum iron (umol/L, median and IQR) | 8.9(8.2,10.2) | 10.6(9.3,13.0) | 0.1 | |
| Leukocyte (10^9/l, median and IQR) | 6.6(6.2,10.3) | 6.0(4.7,7.0) | 0.2 | |
| Lymphocyte (10^9/l,median and IQR) | 1.2(1.0,1.7) | 1.0(0.8,1.4) | 0.2 | |
| C-reactive protein (mg/l,median and IQR) | 5.7(1.8,13.5) | 2.8(1.6,8.9) | 0.3 | |
| ALT (IU/L, median and IQR) | 10.0(8.0,12.0) | 10.0(7.0,15.0) | 0.8 | |
| AST (IU/L, median and IQR) | 13.0(10.0,15.0) | 14.0(11.0,17.0) | 0.8 | |
| ALP (IU/L, median and IQR) | 116.0(88.0,203.0) | 96.5(71.0,124.0) | 0.2 | |

Supplemental Table S2. Comparison between asymptomatic patients and symptomatic patients with COVID-19 during SARS-CoV-2 infection

|  | Asymptomatic patients  N=11 | Symptomatic patients  N=82 | | P |
| --- | --- | --- | --- | --- |
| BMI (kg/m2, median and IQR) | 28.0(22.6,30.7) | 23.9(21.6,25.7) | 0.1 | |
| Monthly averaged pre-HD systolic BP (mmHg, mean ± SD) | 155.0±20.8 | 152.1±15.3 | 0.6 | |
| Monthly averaged pre-HD diastolic BP (mmHg, mean ± SD) | 81.4±16.4 | 79.0±12.0 | 0.6 | |
| KT/V (median and IQR) | 1.6(1.5,1.7) | 1.5(1.3,1.7) | 0.7 | |
| Weekly averaged EPO dose (IU,median and IQR) | 9000(6000,15000) | 9000(6000,11250) | 0.3 | |
| Hemoglobin (g/L,median and IQR) | 109.0(100.0,118.0) | 111.5(106.0,118.0) | 0.5 | |
| Albumin (g/L, mean ± SD) | 35.0±2.8 | 36.3±3.5 | 0.2 | |
| Potassium (mmol/L, mean ± SD) | 4.2±0.5 | 4.5±0.6 | 0.1 | |
| Sodium (mmol/L, mean ± SD) | 136.1±3.0 | 135.4±3.7 | 0.5 | |
| Ferritin (ng/L, median and IQR) | 484.5(235.2,1030.4) | 538.2(352.2,804.9) | 0.5 | |
| Serum iron (umol/L, median and IQR) | 6.9(4.7,11.9) | 9.5(6.9,13.3) | 0.2 | |
| Leukocyte (10^9/l, median and IQR) | 6.2(4.9,7.7) | 5.5(4.3,6.7) | 0.5 | |
| Lymphocyte (10^9/l,median and IQR) | 0.9(0.7,1.1) | 0.9(0.7,1.2) | 0.7 | |
| C-reactive protein (mg/l,median and IQR) | 19.1(5.5,27.9) | 11.3(2.9,45.6) | 0.5 | |
| ALT (IU/L, median and IQR) | 14.0(8.0,30.0) | 10.0(8.0,17.0) | 0.2 | |
| AST (IU/L, median and IQR) | 21.0(14.0,32.0) | 17.0(12.0,22.0) | 0.06 | |
| ALP (IU/L, median and IQR) | 90.0(73.0,231.0) | 82.5(66.0,110.5) | 0.1 | |

Supplemental Table S3. Changes after SARS-CoV-2 infection 2 months in MHD patients with MHD

|  | Before SARS-CoV-2 infection | After SARS-CoV-2 infection 2 months | P |
| --- | --- | --- | --- |
| Monthly averaged pre-HD systolic Bp(mmHg, mean ± SD) | 151.4±15.4 | 151.2±19.6 | 0.9 |
| Monthly averaged pre-HD diastolic Bp(mmHg, mean ± SD) | 78.4±11.6 | 77.4±11.7 | 0.6 |
| KT/V (median and IQR) | 1.5(1.4,1.7) | 1.6(1.4,1.7) | 0.5 |
| Weekly averaged EPO dose (IU, median and IQR) | 7500(6000,9000) | 9000(6000,12000) | 0.03 |
| Hemoglobin (g/L, median and IQR) | 113.0(108.0,120.0) | 112.0(105.0,116.0) | 0.2 |
| Albumin (g/L, mean ± SD) | 39.4±3.2 | 39.2±3.5 | 0.7 |
| Potassium (mmol/L, mean ± SD) | 4.8±0.7 | 4.8±0.6 | 0.8 |
| Sodium (mmol/L, mean ± SD) | 136.5±2.9 | 137.0±3.3 | 0.3 |
| Leukocyte (10^9/l, median and IQR) | 6.2(4.8,7.0) | 6.2(5.0,7.3) | 0.9 |
| Lymphocyte (10^9/l, median and IQR) | 1.1(0.8,1.4) | 1.1(0.8,1.4) | 0.8 |
| C-reactive protein (mg/l, median and IQR) | 2.9(1.6,9.1) | 3.4(1.6,8.5) | 0.8 |
| Predialysis Weight (kg, median and IQR) | 67.7(57.6,77.3) | 66.3(56.8,76.9) | 0.7 |
